# The Impact of Climate Change on Health Services in Low- and Middle-Income Countries: A Systematised Review and Thematic Analysis

**DOI:** 10.3390/ijerph21040434

**Published:** 2024-04-03

**Authors:** Kamar Naser, Zaeem Haq, Bernard D. Naughton

**Affiliations:** 1 School of Pharmacy and Pharmaceutical Sciences, Trinity College Dublin, College Green, D02PN40 Dublin, Ireland; 2 Save the Children St Vincent House, 30 Orange Street, London WC2H 7HH, UK; 3 Centre for Pharmaceutical Medicine Research, Institute of Pharmaceutical Science, Kings College London, London SE1 9NH, UK

**Keywords:** climate crisis, healthcare delivery, low- and middle-income countries

## Abstract

**Aim**: The aim of this study was to assess the impact of climate change on health services as categorized by the WHO’s Building Blocks for creating Climate-Resilient Health Systems. **Objective**: The objective was to conduct a systematized review of the published literature concerning the impact of climate change, using a thematic analysis approach to address our aim and identify areas for further research. **Design**: A search was conducted on 8 February 2022 using the Embase and PubMed research databases. Peer-reviewed scientific studies that were published in English from 2012 to 2022, which described at least one report concerning the impact of climate change on health services in LMICs, were included. Studies were organized based on their key characteristics, which included the date of publication, objective, method, limitations, participants, and geographical focus. The Mixed-Methods Appraisal Tool (MMAT) was used to assess the risk of bias in the included studies. **Results**: Twenty-three studies were included in this review. Five areas of health services which align with the WHO building blocks framework were impacted by climate change. These health service areas included: (1) Service Delivery, (2) Human Resources, (3) Health Finance, (4) Healthcare Products and Technology, and (5) Leadership and Governance. However, research concerning the impact of climate change on health information systems, which is part of the WHO building blocks framework, did not feature in our study. The climatic effects were divided into three themes: meteorological effects, extreme weather events, and general. The research in this study found that climate change had a detrimental impact on a variety of health services, with service delivery being the most frequently reported. The risk of bias varied greatly between studies. **Conclusions**: Climate change has negatively impacted health services in a variety of different ways, and without further actions, this problem is likely to worsen. The WHO building blocks have provided a useful lens through which to review health services. We built an aligned framework to describe our findings and to support future climate change impact assessments in this area. We propose that further research concerning the impact of climate change on health information systems would be valuable, as well as further education and responsible policy changes to help build resilience in health services affected by climate change.

## 1. Introduction

The World Health Organization (WHO) states that climate change is humankind’s single biggest health threat [1]. In 2022, the world saw the highest global temperatures for over 100,000 years. Considering global fossil fuel investment increased by 10% in 2022, reaching over USD 1 trillion [2], action against climate change does not appear to be moving quickly enough. Low- and middle-income countries (LMICs) suffer the greatest risks from climate change, despite emitting relatively low levels of greenhouse gases. Fourteen percent of global CO_2_ emissions come from LMICs, while the remainder comes from high- and upper-middle-income countries [3]. LMICs are not only the least equipped to defend themselves, but they also reap the least benefit from the economic and technical advancements that have contributed to climate change [4]. The Lancet Commission on Health and Climate Change, established in 2015, publishes annual assessments of the present situation. They found that climate change is already having an impact on health, with future forecasts indicating an unacceptably high and perhaps catastrophic danger to human health. In November of 2023, the most recent revised report was released. The report concluded that the world has made little progress in protecting individuals from the adverse health effects of climate change, and the prevalence of climate-sensitive infectious diseases continues to rise. The transmission season for malaria, for example, is lengthening in several regions, with the biggest increase occurring in the African highlands for Plasmodium falciparum (0·61 months) and in the South and Central American highlands for P vivax (0·8 months) [2]. Temperature rises have caused a slew of issues, including an increase in cardiovascular and respiratory problems. Wildfires and droughts have also increased dramatically according to the Intergovernmental Panel on Climate Change (IPCC) [5]. Such calamities could have disastrous consequences on health services, whose main mission is to maintain human health. Global warming and climate change are also increasing the risk of illness, which is increasing the demand for health services, which are not equipped to handle the increase. Although studies regarding climate change and health services have been conducted, there is no comprehensive evaluation of the available literature on this matter in LMICs. Research into climate change in higher-income countries is driven by government policies to reduce carbon emissions, many of which have adopted by large, multinational companies. However, lower-income countries often feel the ill effects of higher-income countries’ carbon emissions much more. To make the situation worse, lower-income countries have fewer resources to allocate to deal with the effects and prevention of climate change [2]. This inability to manage the effects of climate change can lead to flooding, landslides, and drought, which have knock-on effects on health and access to healthcare services. To make matters worse, low and medium Human Development Index countries contained the highest proportion of cities not intending to undertake a climate risk assessment in 2021 [2].

The definition of health services which we propose is ‘The art and science of preventing disease, prolonging life and promoting health through the organized supply of medical care’, and is based on the definition of public health [6]. We structured our impact assessment approach according to the WHO building blocks framework for building climate-resilient health systems [7]. These WHO building blocks are proposed as a way to help strengthen the health system of a country in different ways [8]. Their goals or outcomes are to improve health, improve efficiency, improve responsiveness, and improve social and financial risk protection [8]. In addition to our primary aim of understanding the impact of climate change on health services, this study aimed to understand if the WHO building blocks framework was a useful lens to see health services through for the purposes of a research-based impact assessment. The objectives were to conduct a systematized review to identify, and then conduct a thematic analysis of, the core dimensions of healthcare services and the impact of climate change on these dimensions in low- and middle-income countries. Without understanding and outlining the current research concerning the main climate change impacts in these regions, it is difficult to plan future research and allocate resources to effectively tackle those impacts. These building blocks are important to strengthen health systems, but it is unclear how they are affected by climate change and what other dimensions of these building blocks are also important. The outcomes of this study could help NGOs and healthcare providers to appreciate the current health service challenges in other countries and support preparations for similar climate changes in their regions. It is hoped that a review of the data shedding light upon climate change’s impact on health services in LMICs may raise awareness within low- and medium-HDI cities and hasten their prevention and response to climate change.

## 2. Methods

A systematized review is one which fulfills many of the criteria of a systematic review. It is therefore more rigorous than a scoping review but, due to a lack of resources, may not include two reviewers at each stage of the review process. This approach was taken to ensure the review was as rigorous as it could be given the available resources. In this case, a second reviewer was not employed during all steps of this process and only two research databases were analyzed. This study was constructed using the PICO framework. The population was those in low- and middle-income countries, the exposure was climate change, there was no control, and the outcome was the delivery of health services. Preferred Reporting Items for Systematic Reviews and Meta-Analysis (PRISMA) 2020 was used to report our findings as a reporting guideline [9]. Patient and public involvement was not sought for this study.

### 2.1. Eligibility Criteria and Rationale

The article inclusion criteria (Table 1) were as follows:

(1) Published between the years 2012 and 2022, to focus on contemporary data.
(2) Academic peer-reviewed articles to ensure academic rigor.
(3) Published in English, as this is the most widely used language for international research publishing.
(4) Full article availability.
(5) Reported at least one impact of climate change on health service(s) in a low- or middle-income country.

### 2.2. Search Strategy

A thorough search of the international literature was undertaken using the EMBASE and PubMed databases. These were selected as the most significant international medical and healthcare publications are indexed in these databases. A combination of free text, MeSH, and Emtree terms (Table A1) were used to identify articles regarding the impact of climate change on health services in LMICs. A comprehensive breakdown of how the keywords were identified (Table A1) and how the search strategy was carried out (Table A2) is contained in the Appendix A. The search was narrowed down even further by using the following filters: (1) date: 2012–2022; (2) only in English; (3) only studies affecting human health; (4) and full text (5).

The authors of this review developed the search technique. All citations identified from the database searches were uploaded to Covidence, a web-based software platform that streamlines the production of systematic reviews and other research reviews that require screening citations and full text, an assessment of the risk of bias, or the extraction of study characteristics and outcomes [10]. Endnote was the reference management tool employed to remove duplicates. 

### 2.3. Selection of Studies

The first author of this systematic review screened the titles and abstracts. Studies that did not match the criteria for inclusion were excluded. The author next retrieved and examined the full-text articles, deciding whether to include them based on the previously established selection criteria. The first and third authors then separately screened all articles according to the inclusion and exclusion criteria. In this case, there were no disagreements regarding the inclusion criteria.

### 2.4. Data Extraction

Using Excel and a Covidence data extraction form, the first author extracted the following data: the study design, the year of publication, the geographical area of interest, participant description, the method, the climatic focus, and reported impacts on health services. The information explored in full-text publications was used to create climate change categories.

### 2.5. Thematic Analysis 

Full-text publications were thematically analyzed to familiarize the authors with the manuscript contents and recurring themes regarding the impact of climate change on health services. The goal was to assess existing research concerning the impact of climate change on health services as categorized by the WHO building blocks framework and to develop an aligned framework to describe our study results and facilitate the performance of future impact assessments concerning climate change and health services. We used the regularly occurring concepts to define themes and subthemes. Supported by the WHO building blocks framework, a preliminary framework was evaluated using a subset of the data to identify areas for improvement and make any necessary changes. The WHO Health System conceptual framework was used to guide the analysis [8] and was then challenged against our data set. The WHO framework consists of six building themes—Service Delivery, Health Workforce, Financing, Leadership and Governance, Healthcare Products and Technologies, and Health Information System (HIS) [7]. This framework helped us summarise data in a way that achieved the study’s aim and helped us to develop a new framework that is suitable for guiding this study and future impact assessments. 

### 2.6. Risk of Bias Assessment

The risk of bias was assessed using a revised Mixed-Methods Appraisal Tool (MMAT), which is a critical appraisal tool that is designed for the appraisal of systemic mixed study reviews [11]. 

## 3. Results

### 3.1. Articles Identified

**Figure 1:**
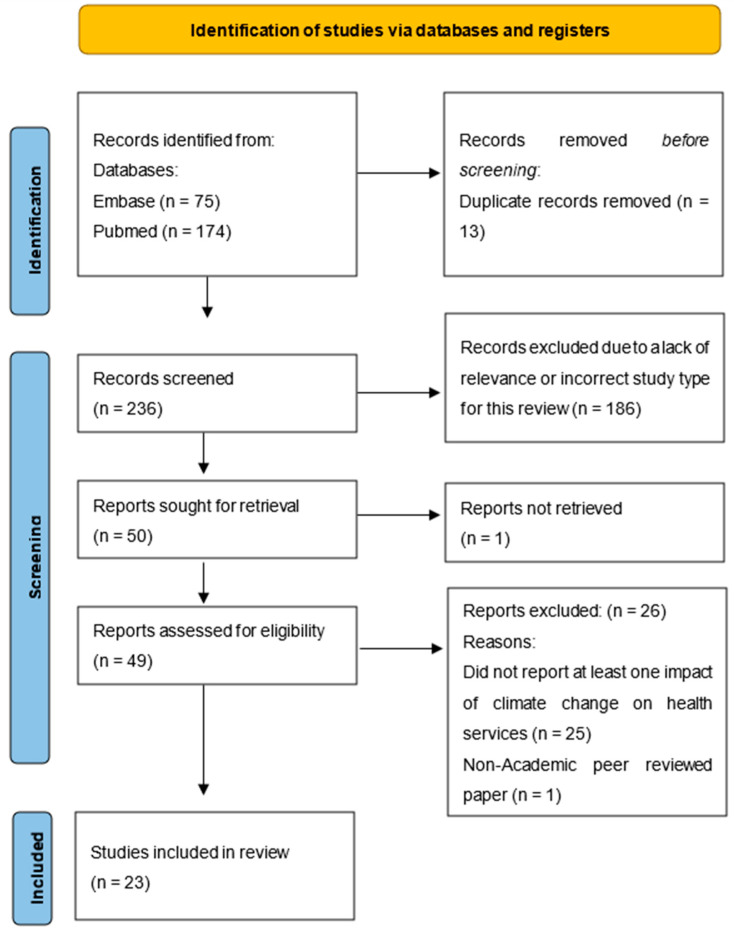
The Prisma flow chart for articles included in this review.

The initial search yielded a total of 249 studies. Duplicate papers were removed (n = 13). The titles and abstracts of 236 studies were reviewed. A total of 186 papers did not fulfil the initial inclusion criteria during the title and abstract screening process; therefore, 50 studies were selected for full-text examination. After reading the full papers, a further 27 studies were excluded as they did not fulfil the inclusion criteria, as they did not report at least one impact of climate change on health service(s) in LMICs, they were not scientific peer-reviewed studies, or the full text was not retrievable (n = 1). Twenty-three studies were included for assessment, as indicated in the PRISMA diagram in Figure 1.

### 3.2. Study Descriptions

**Figure 2:**
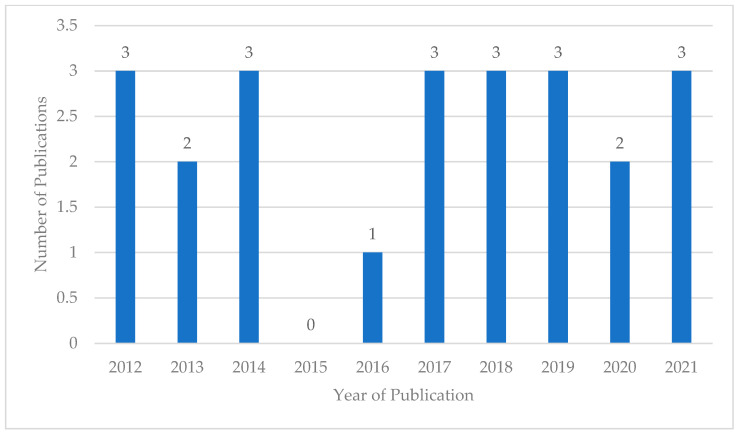
Number of included articles by year of publication.

All the articles and their characteristics are contained in Table A3. From 2012 to 2021, 1–3 papers were published annually; 2015 was the only year in which no papers were published. As 2022 had not yet elapsed at the time of the study preparation, that year is not included in Figure 2. 

Twenty-three articles were analyzed in this study. Nine were qualitive, eight were quantitative, three were mixed-methods, and three were reviews. Most of the participants in the study were professionals from different disciplines, healthcare or climate change experts, or government or NGOs representatives (n = 8) [12,13,14,15,16,17,18,19], or involved local residents (n = 5) [20,21,22,23,24]. In the rest of the studies, either no participants were enrolled (n = 7) [25,26,27,28,29,30,31] or locals and experts were enrolled together (n = 4) [32,33,34] (Table A3).

In terms of geographical focus, some studies focused on specific countries (n = 19) (one study for each of Barbados, Dominica, Kiribati, Fiji, India, Pakistan, Singapore, Vietnam, China, Russia, Ethiopia, Zambia, Ghana, and Macedonia, two for Brazil, and three for Bangladesh), regions (n = 2) (one each for the Horn of Africa and Caribbean Small Island Development), or income levels (n = 2) (two on low- to middle-income countries). (Table A3).

### 3.3. Risk of Bias Assessment

The bias of the studies was analyzed using the Mixed-Methods Appraisal Tool (MMAT) method. All the studies met the inclusion criteria. The MMAT could not be used to evaluate three studies [12,14,17] as they were secondary research. Qualitative interviews were a common technique for gathering data, but only a few of them examined the influence the interviewer may have on the individual being interviewed; therefore, interviewees may have been subject to recall bias. (Table A4, Table A5 and Table A6).

### 3.4. Climate Impacts

The climatic effects were divided into three categories; meteorological effects (n = 4) (temperature changes, humidity, and precipitation); extreme weather events (n = 11) (floods, storms, cyclones, and drought); general (n = 8) (these studies focused on climate change in general rather than a specific climate change impact). (Table A3).

### 3.5. Health Services

**Figure 3:**
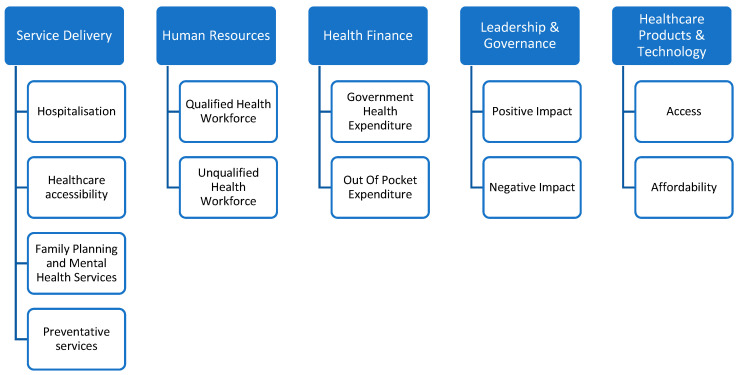
The themes and subthemes of health services impacted by climate change.

Thematic analysis produced five health service themes affected by climate change and aligned with the WHO building blocks. Some papers included multiple themes. These were as follows: (1) service delivery (n = 21) [12,13,14,15,16,17,19,20,21,22,24,25,27,28,29,30,31,32,33,34], (2) human resources (n = 6) [13,15,16,22,23,34], (3) health finance (n = 10) [13,15,16,17,19,21,22,23,31,33], (4) healthcare products and technology (n= 3) [15,22,23], and (5) leadership and governance (n = 12) [13,14,15,16,18,19,20,21,25,28,31,33]. The Health Information System (HIS) pillar/theme lacked sufficient data, i.e., none of the included studies explored this theme. The five themes aligned well with the WHO health system framework. Figure 3 outlines the health service themes and subthemes that are most commonly impacted by climate according to the literature.

### 3.6. Service Delivery

Among the themes reviewed in this study, service delivery was researched the most, with 19 papers covering this theme. The reported impacts on health services were identified and outlined as follows: (1) hospitalization (n = 6), (2) access to healthcare (n = 10), (3) Family planning and mental health services (n = 6), (4) preventive services (n = 6). Some papers included multiple themes. A summary of the themes and subthemes is presented in Figure 3.

#### 3.6.1. Hospitalization 

Studies have shown that climate change is associated with many health outcomes that influence hospital admissions. The Dengue epidemic in Barbados [25], and the malaria outbreak in Ethiopia [28] are examples of how extreme weather effects could cause a surge in hospitalization. Meteorological changes have also been linked to an increase in hospital admissions for cardiovascular [14,17] and respiratory illnesses [30]. Extreme weather events and meteorological changes were also found to be associated with an increase in hospitalizations for diarrheal infectious diseases [29].

#### 3.6.2. Health Care Accessibility 

Evidence from the studies suggests that communication was hindered and accessibility to health services was hampered during extreme weather occurrences. In Bangladesh [34], where boats were the only way to get to the hospital during a storm, these trips were difficult to arrange and time-consuming, and boatmen were scarce or charged a high fee for emergency boat transportation. In Vietnam [15], accidents were the most prevalent causes of persons needing to seek healthcare during storms and floods. However, owing to building damage, power outages, or insufficient professional personnel, medical emergency services, particularly surgical procedures, and referral systems were not always accessible during storm and flood seasons.

Studies have also shown that climate change had a greater impact on certain groups of people, including indigenous people, women, and people in remote or rural areas [21,22]. Nomadic herders in Russia, who traveled mainly on foot, or using sleds and snowmobiles, found themselves a long way from health services due to the emergence of new lakes, streams, and shortened winter periods [24]. The Kalinago community in Dominica also had limited capacity to cope with climate change [12] and the mobility of Kenya’s nomadic pastoralists made accessing health services very challenging for these groups.

Migrants were also among those groups who had difficulty accessing health services. In the Horn of Africa [16], droughts have had devastating impacts on agriculture, causing harvest failure, food shortages and increased food prices. The absence of adequate food hindered economic growth, exacerbated conflict and political instability, and prompted internal or cross-border migration. Migrants, particularly those who were undocumented, faced several barriers to treatment, including high fees, denial of care, and discriminatory practices. On the other hand, some research found that using planned migration and relocation as an adaptive technique could improve accessibility, as observed in Fiji [20], China [19], and Ghana [33].

#### 3.6.3. Family Planning and Mental Health Services 

A study in Zambia suggests that households in climate-change-affected regions struggled to find steady or adequate jobs, which ultimately led to an out-migration trend, increased rates of early marriages, and exchange sex. Marital relationships were challenged, and many women were overburdened by a perceived imbalance in household and income-generating responsibilities. Women’s and men’s fertility intentions changed in tandem with household income, increasing the demand for family planning services, which were in short supply and inadequate [32]. Climate change also increased people’s susceptibility to developing mental illness or exacerbated existing mental illness; however, mental health resources were scarce in all studies that explored this matter [13,14,16,19,31]. The Kiribati government has classed mental and reproductive health care as low priority and not as crucial as other services [19].

#### 3.6.4. Preventive Services 

The quality of the preventive measures was inadequate. Several of the studies found that surveillance systems, such as early warning systems, were either not in place or not included in emergency preparations [13,25,27,31,33], although one study found that China had a good early warning system for floods [19].

### 3.7. Human Resources

This element of health services included research concerning both qualified and unqualified human resources. Some studies suggest that, due to the lack of health services and transportation in rural areas during climate change incidents, or due to the poor services and bad infrastructure, a significant number of people resorted to unqualified practitioners to deal with their climate-health-related concerns or their health needs in general [22,23,34]. Often, health workers in public sector health facilities were unable to provide the desired level of service due to their low level of education and understaffing. [16], or were ill-trained in how to manage during climate or natural disasters [13,15].

### 3.8. Health Finance

The theme of health finance contained two subthemes. These were (1) government health expenditure and (2) out-of-pocket payments expenses.

#### 3.8.1. Government Health Expenditures 

The studies revealed that health expenditure increased because of climate change [13,31]. Moreover, governments failed to adequately budget for climatic change and health emergency plans. The lack of funds made it difficult to implement effective early warning systems or other adaptation measures that are necessary to improve the preparedness of health facilities [15,33]. It was also shown that the healthcare agenda was set by donors, which do not always reflect the population’s needs. This assistance dependency poses a problem for low- and middle-income countries [16].

#### 3.8.2. Out-of-Pocket Payments (OOP) 

Climate change has put people’s health in jeopardy, causing a rise in demand for health services and, as a result, greater healthcare costs. Patients had to pay for healthcare, which resulted in a rise in out-of-pocket payments. The studies highlighted that some health systems rely extensively on OOP [21,22], placing vulnerable groups under more pressure [16] and perhaps preventing them from receiving healthcare [17]. Access to healthcare, medication adherence [23], and quality of life are all hampered by high rates of OOP. Sometimes, high costs were incurred to receive low-quality healthcare from inexperienced providers [22] due to factors such as the lack of availability of qualified practitioners during disasters. Introducing health insurance schemes is a great tool to finance health care problems and an effective measure to combat this problem, as seen in Ghana [33]; however, evidence from other studies suggests that there are some countries that have no health insurance schemes or insurance policies to cover the risks arising from climatic change [19], nor is there an NGO initiative to support the high out-of-pocket payments and health expenses [22]. 

### 3.9. Leadership or Governance 

Leadership and governance of health services has been required in response to climate change. Through the introduction of policies, laws, and plans, organizations and governments have attempted to mitigate the ill effects of climate change and these actions have had direct, indirect, positive, and negative impacts on health services [13,14,15,16,18,19,20,21,25,28,31,33]. In some cases, these climate-change-linked policies did more harm than good, as seen in Ethiopia, where the government tried to combat the effect of droughts by constructing dams and irrigation systems. These changes benefited the agricultural sector and helped boost rice farming, which, in turn, had an indirect effect, altering environmental conditions and improving the circumstances required for mosquito breeding. Malaria transmission surged as a result, exacerbated by population migration, spurring the demand for health services that were not equipped to handle the increasing demand [28]. Barbados’ government also attempted to address water scarcity issues caused by drought by enacting a legislation requiring the construction of rainwater storage containers under new buildings. Those containers served as an ideal larval environment for Aedes aegypti, increasing their transmission and consequently increasing hospital admissions [25]. According to some studies [19,20,33], there have been instances where such policies have proven useful, as they made healthcare more accessible and reduced morbidity, but they still faced many problems. 

### 3.10. Healthcare Products and Technologies

The impact of climate change on healthcare products and technologies has only been explored in three studies, which highlight the importance of access and affordability. A study found that health facilities had access to standard protocols, but not to emergency protocols [15]. In India [23] and Bangladesh [22], people were self-medicating or relying heavily on advice regarding medication from unqualified practitioners to deal with the previously discussed climate-sensitive diseases due to the lack of availability of trained healthcare providers or their high costs. Climate change has been linked to the health issues discussed in this paper, increasing the demand for healthcare products. However, medicines were found to be expensive, and many people were not able to afford them; as a result, there were problems with compliance, leading to a slew of issues such as antibiotic resistance because of people not purchasing the full course of antibiotics [23].

### 3.11. Climate Change Impact Assessment Framework

The building blocks framework guided the development of this new framework (Figure 3). This framework demonstrates how health services can be impacted by climate change, with some changes to the WHO framework to support a clearer and more inclusive impact assessment approach. These changes included replacing the terms health workforce with human resources, health system financing with health finance, and medicinal products, vaccines, and technology with healthcare products and technology. There were no examples of research concerning climate change and health information services found in this systematised review to support the inclusion of this dimension as part of this framework. In addition, subthemes for each of the adjusted building blocks were included to facilitate further, more detailed, impact assessments in this research area as they emerged in the literature.

## 4. Discussion

This systematized qualitative review was designed to highlight examples of how climate change impacts health services, as seen through the WHO building blocks lens, by exploring the relationship between both in LMICs and mapping the characteristics and findings of the studies that explored those relationships. In doing so, a framework was created to outline the impact of climate change on the different dimensions of health services and the different ways in which these health service dimensions were affected. Multiple key findings emerged from this process.

### 4.1. A Lack of Evidence in LMICs

First, heterogeneous studies were included in this study, with qualitative research being more prevalent than quantitative studies, as well as mixed-methods studies or reviews. Initially, we intended to rely only on primary research, but the lack of available data led us to also include secondary research papers. There were three secondary studies, all of which were reviews of climate change and health Vulnerability and Adaptation Assessments (V&As). Although reviews and vulnerability studies are useful and have contributed to the Lancet Countdown and The Intergovernmental Panel on Climate Change (IPCC) reports, this suggests that there is limited primary research in the area of climate change and its impact on health services. The secondary research studies we found provided evidence-based information regarding current and future risks to health, vulnerable populations, and effective adaptation options. We suggest that the information generated from such assessments could continue to contribute to international scientific reports, such as studies by the IPCC and Lancet Commission on Health and Climate. They could aid officials in navigating these challenges and by developing effective measures to overcome them. However, further primary research studies could be funded to add additional weight to these reports. Therefore, we suggest that more countries should undergo V&As regularly, preferably on a yearly basis, and encourage further investment into primary research to assess the impact of climate change on health services as, unfortunately, most countries in this study are not doing so [35,36]. 

In line with previous studies [37,38], our study showed that there was a relationship between climate change and the number of hospital admissions. According to Bishop-William et al. [39], there are many available studies investigating this association; however, most of them were focused on high-income regions and the ones in low-income regions were locally focused and varied in terms of health outcomes, which aligned with our results. Although little is known about the overall impact of climate change on hospital admissions for all diagnoses, the available information generally supports the conclusion that climate change negatively affects human health, which will ultimately cause hospital admission rates to increase. The impact of climate change on hospital admissions will vary globally, so there is no single general solution that will be effective across all countries and contexts. Therefore, regionally specific studies are needed to characterize the impacts of climate change on health outputs and healthcare at local levels and in specific contexts [39,40,41,42].

### 4.2. Direct versus Indirect Policy Impact and Responsible Innovation

Another key issue identified in this study was a lack of distinction or classification and focus on the direct and indirect impacts of climate change policy on health services. It is important to recognize the complexity of leadership or governance decisions and how they can have both direct and indirect effects on health services. Impact assessment is a complicated process and what appears to be a positive policy intervention through one lens, may have direct or indirect and less favorable outcomes, as seen in the two government decisions to manage the effects of climate change that led to an increase in Malaria in the case of Ethiopia and an increase in Aedes aegypti larvae in Barbados [25,28]. These interventions appeared to lack a sense of responsible innovation, which is an approach which includes anticipatory governance, inclusivity, reflexivity, and responsiveness. Had the governments practiced anticipatory governance, included the correct stakeholders, asked the right questions and reflected upon their own misconceptions, and acted upon stakeholder input, they may have identified some of the risks linked to their decisions [43,44,45]. Furthermore, when there is an absense of data to support evidence-based policy decisions, a responsible innovation or leadership approach could be used to ensure the right stakeholders are involved in policy changes to avoid direct and indirect, unanticipated, negative impacts [43,44,45]. 

### 4.3. Early Warning Systems

The introduced means of preparing for climate-related health effects include early warning systems (EWS). These are adaptive measures for climate change, using integrated communication systems to help communities prepare for hazardous climate-related events. Successful EWS save lives, jobs, land, and infrastructures, and support long-term sustainability [46]. It was estimated that EWS have saved hundreds of lives every year in Europe and prevented disaster losses of up to EUR 2.7 billion. According to the WHO, it is difficult to estimate the existing availability of EWS [24]. There were some countries that were able to develop efficient systems, but there were others without basic observation systems, which made the implementation of EWS impossible. It is important that effective EWS are established for climate-sensitive health risks. Even though Early Warning Systems are beneficial, simply delivering early warnings is insufficient. Climate data could be officially included in national and sectoral planning. Health experts could be trained to read and communicate results, and partnerships with stakeholders and localities could also be strengthened.

### 4.4. Climate Change and Mental Health

Unsurprisingly, studies have suggested that climate change negatively impacted mental health because it aggravated social and economic problems. Studies that examined the impact of climate change on mental health in this review were primarily focused on the direct impact of experiencing extreme weather events. Unemployment, forced migration, stress, and unhealthy coping mechanisms as a result of extreme weather events also contribute to mental health problems. For example, in Eritrea, droughts have prompted an upsurge in migration to neighboring countries such as Ethiopia. Since the start of 2017, more than 4500 Eritrean refugees have entered Ethiopia according to the International Organisation for Migration (IOM) [47]. Even though both nations have comparable cultures, disagreements and arguments were bound to arise, causing migrants to be socially ostracized, and therefore contributing to mental health problems. However, it has been established by many researchers and healthcare professionals that climate change could also directly affect mental health (e.g., eco-anxiety). They have also found that extreme weather events and meteorological changes can both negatively affect mental health. Increased temperatures have been linked to an increased risk of anxiety, mood disorders, and suicide, as well as thyroid malfunction, which can lead to mood disorders [48,49,50]. Despite the growing demand for mental health services and the increasing awareness and availability of research on the relationship between mental health and climate variability, mental health services are still scarce. Physical needs are not being addressed sufficiently during climate change disasters, so it is no surprise that mental health concerns remain largely unaddressed. Many of these problems result from the lack of political pressure to treat mental health seriously, the absence of mental health legislation, and donor organizations’ tendency to focus on more clearly quantifiable outcomes. 

### 4.5. The Role of the Healthcare Professional

In the face of climate change, healthcare providers play an important role in protecting people and ensuring the functioning of the healthcare system. According to Dupraz et al., health professionals are vital for raising awareness and driving policy initiatives [51]. A survey conducted in the US found that physicians and nurses were the most trusted sources of information about climate change’s effects on health [52]. Having said that, our study has shown that poor training, a lack of education, and a shortage of qualified health professionals makes it difficult for them to play their role in addressing climate change’s impacts, as their attention is largely focused on healthcare delivery and they may not have the time and capacity to consider the direct and indirect impacts of the climate on healthcare. A lack of education and a lack of capacity to consider the climate–health service link may mean that the root cause of these climate-related illnesses remain unaddressed. The WHO reports that the greatest shortage of health workers is found in low- and middle-income countries. Current trends in health worker production and employment will not have a sufficient impact on the needs-based shortage of healthcare workers by 2030 [53]. Consequently, healthcare providers will have to work even harder to make up for the demand–supply gap, which is exacerbated during crises such as floods and storms. They can easily become overburdened and stressed, with poor health outcomes, unmet healthcare demands, and higher healthcare expenses as a result. Climate change has also caused people to seek health services from unqualified practitioners. This problem was more pronounced among vulnerable populations. Our findings suggest that climate change exacerbated inequalities in access to healthcare among certain groups of people. There is no doubt that the effects of climate change will be felt by all, but certain groups will be disproportionately affected, including women, migrants, indigenous peoples, and those in rural or remote areas. Jesdale et al. [54] obtained similar results. A complicated issue, such as the health workforce imbalance, requires a well-thought-out strategy and policy. Continuity of care can only be ensured through health planning. To address these problems, decision-makers could identify which areas are underserved, forecast future health personnel needs, and develop policies to accommodate them [55]. Climate and health linkages should be covered in the training provided to practitioners with experience in the field, as well as obtaining a better understanding of climate services for health, how to use them in the case of an emergency, and how to communicate the climate’s effects on health to local communities. Healthcare professionals could also be shown how to use geographic information systems to view and analyze biological, vector, and climatic data. It is important to point out that, in theory, this problem could be solved; however, this is far more complex in reality, especially in resource-constrained settings.

### 4.6. Cost

This review has suggested that both OOP and government expenditures have increased because of climate change. Patients in developing countries spend half a trillion USD each year out of their own pockets to receive health services [56]. The total OOP spending increased at least twice as much in low- and middle-income countries during 2000–2017 [57]. Costs have acted, and will continue to act, as access barriers to many people. In the 2020 National Health Interview survey, 1 in 11 adults in the US reported delaying or going without healthcare due to cost reasons. The situation in low-income regions is undoubtedly worse. Medication use has also been negatively impacted by high prices. Drug costs were about 68 percent of OOP payments in lesser-developed countries [57]. Forgoing treatment due to high costs could increase the risk of longer-term deterioration and could push some people into extreme poverty. There is no easy solution to this problem; however, well-designed policies and strategies can help countries to reduce their OPP. OPP payments could be reduced by drug ceiling rates and insurance coverage; however, payments should be based on household ability and income to ensure they are efficient and fair. For example, in Palestine, insurance systems were found to be equitable because they took people’s abilities into consideration [12].

### 4.7. Appetite for Action

The studies in this paper demonstrate that governments around the globe recognize the importance of responding to climate change, which has caused many policies and plans to be developed to address climate change. Many of these policies could have an impact on health services. According to Lavis et al., the implementation of such policies is often ad hoc or fragmented [13], which is consistent with the findings of this review. Other factors which are likely to affect the appetite for action are political stability, robust democracies, and socioeconomic advancement [58,59]. Poor policy management practices can also unnecessarily lengthen the policy development and adoption process. A lack of funds, human resources, and coordination make implementing policies difficult. Not only must a policy be introduced, but it must also be implemented, monitored, and managed efficiently. 

Although the WHO building blocks could be improved when it comes to more specific contexts [60], they acted as a positive lens through which to see health services. These building blocks helped us to create an aligned framework to clearly describe the impact of climate change on health services. However, in order to assess the impact of climate change on health systems moving forward, it is important to be cognisant of the differences between HICs and LMICs. HICs have access to far more sophisticated health information systems; therefore, impact assessments of climate change on health systems in LMICs are unlikely to always include a strong focus on health informations systems. We suggest further research to continue the impact assessment of climate on health services, and we suggest that our framework (Figure 3) could provide a positive approach to conducting such future impact assessments. 

### 4.8. Strengths and Limitations

* This systematised literature review offers a robust and reliable overview of the current state of play in climate change and research on access to health services.
* To the best knowledge of the authors, this is the first study of its kind on this topic.
* The research was undertaken using two databases. Despite these being extensive resources, by not including more databases, the authors may have omitted some important papers.
* Due to resource availability, not all analysis steps were performed by two reviewers.
* Many of the studies contained in this systematic review did not control for other factors in the research setting, which could include conflict, health service fragility, socioeconomic factors, or chronic under-investment in health systems in LMICs.

## 5. Conclusions and Future Research

Climate change places the components of the health system that contribute to the system’s strength in jeopardy, and this problem could grow more problematic as the effects of climate change worsen. This study outlined the impact of climate change on the WHO building blocks of health services in LMICs. We identified service delivery, human resources, health finance, leadership/governance, and healthcare products or technology, as well as various subthemes, to describe how health services have been impacted by climate change. Climate-related planning, and the introduction of early warning systems were identified as useful management approaches.

Climate change also appears to be affecting mental health. Improving healthcare professional education concerning climate change is likely to play a crucial part in managing this issue moving forward. Despite the cost challenges seen in LMICs, there does appear to be some appetite for action, and the framework emerging from this study could be used to guide further research and impact assessments of climate change on health services. 

The articles in this study found that climate change has an impact on healthcare services; however, this relationship is very complex, containing both direct and indirect effects. One high-level approach to managing the complexity of climate change is through the adoption of responsible innovation principles. Despite the current literature covered in this study, there is a lack of primary research focusing on the impact of climate change on health information systems. Therefore, further research studies in this area would be valuable. Future studies could also fill knowledge gaps by exploring ways to estimate the impact of climate change on health services and continuing to identify suitable mitigation measures. 

## Figures and Tables

**Table 1:** Study inclusion and exclusion criteria.

Inclusion Criteria and Explanation	Exclusion Criteria
Studies published between the years 2012 and 2022 To ensure we identified contemporary data	Studies which were published before 2012 or after 2022
Studies which were published in English. English is the most widely used language in academic publications	Studies published in a language other than English.
Accessible full articles To facilitate data analysis	Inaccessible full articles
Studies that reported at least one impact of climate change on health service(s) in LMICs To align with the study objectives	Studies that did not report at least one impact of climate change on health service(s) in LMICs or studies which reported the impact of climate on a specific condition
Academic peer-reviewed papers To ensure the studies were rigorous	Non-academic or non-peer-reviewed papers

## Data Availability

All relevant data are provided in this article. For further information, please contact the corresponding author.

## Appendix A

**Table A1:** Keyword identification.

	Climate Change	Health Services	LMIC
**Google search**			List of LMICs according to The World Bank. Low- and middle-income|data
**Synonyms**	Climate variability Greenhouse effect	General medical service Medical commission Health practice * Hospital medical system	Lower-middle-income countr * Low-income countr * Middle-income countr * Middle-income economy countr * Low-income economy countr *
**Emtree**	Meteorological phenomena	Emergency health service Rescue personnel Medical service * Health service * Medical information System	
**MeSH**	Ecological and environmental phenomena Climate change * Global warming	Emergency medical service communication systems Emergency medical services Pharmaceutical services Clinical laboratory services Reproductive health services Maternal–child health services Reproductive health services Health care costs Delivery of healthcare Patient care management Hospital medical system	Developing countr * Least developed countr * Less developed countr * Underdeveloped nation * Underdeveloped countr * Developing nation * Less developed nation * Third world countr *

**Table A2:** Search strategy.

**Search 1:**	Climate variability, greenhouse effect, meteorological phenomena, ecological and environmental phenomena, climate change, global warming
**Search 2:**	General medical service, medical commission, health practice, hospital medical system, emergency health service, rescue personnel, medical service, health service, medical information system, emergency medical service communication systems, emergency medical services, pharmaceutical services, clinical laboratory service, reproductive health services, health care costs, delivery of healthcare, patient care management, hospital medical system
**Search 3:**	Lower middle income countr *, Low income countr * Middle-income country *, Middle-income economy countr *, Low-income economy countr *, Developing country *, Least developed countr *, Less developed countr *, Underdeveloped nation *, Underdeveloped countr *, Developing nation *, Less developed nation *, Third world countr * World Bank’s list of LMICs (The full list of countries is available in the appendix figure…)
**Search 4:**	S1 AND S2 AND S3

**Table A3:** Summary of included studies.

Study Title	Type of Study	Year of Publication	Geographical Interest	N	Participants	Method	Climate Change Focus
Schnitter et al. [12]	World Health Organisation (WHO) assessment	2018	Dominica	N/A	A group of climate and health experts, as well as representatives of NGOs and government agencies	Secondary research	General
Stewart-Ibarra et al. [13]	Qualitative description	2019	Dominica Barbados	73	Stakeholder—public decision-makers, practitioners from the climate and health sectors at the regional level	In-depth interviews, survey, workshop.	Extreme weather—drought
Lowe et al. [25]	Modelling study—quantitative study	2018	Barbados	N/A	No participants	Distributed lag nonlinear models (DLNMs) coupled with a mixed-model framework.	Extreme weather—drought and meteorological—rainfall
McIver et al. [14]	Review of vulnerability and adaptation assessment to climate change—secondary research	2014	Kiribati	N/A	A group of climate and health experts, as well as representatives of Non-Governmental Organisations (NGOs) and government agencies	Secondary research	General
McMichael et al. [20]	Case study—qualitative study	2021	Fiji	N/A	Locals (residents in the village of Vunidogoloa)	In-depth interviews, group discussion	Extreme weather—water-level rises
Abdullah et al. [34]	Perspective of case studies—qualitative study	2019	Bangladesh	12	Locals (residents, village doctors, and traditional birth attendants in Khaliajuri)	In-depth interviews, group discussion	Extreme weather—floods
Kabir et al. [21]	Cross-sectional study—qualitative	2016	Bangladesh	6720	Locals (head of households—men)	In-depth interviews, group discussion	Extreme weather—floods, storms
Haque et al. [22]	Cross-sectional study—mixed-method study	2013	Bangladesh	450	Locals	Interview, administered questionnaires. Literature review.	extreme weather—floods, storms
Sahoo et al. [23]	Qualitative description	2012	India	53	Locals (people in Malkangiri and Khurda from various backgrounds, with a range of ages, genders, educational levels, and occupation)	In-depth interviews, group discussion,	General
Malik et al. [26]	Quantitative study	2012	Pakistan	N/A	NO human participants	Climate change vulnerability index	General
Aik et al. [27]	Time series analysis—quanititative study	2020	Singapore	N/A	No human participants	Time series analysis	Meteorological—relative humidity, increase in temperature and rainfall
Van Minh et al. [15]	Cross-sectional descriptive study— mixed-method study	2014	Vietnam	24	Health experts, civic leaders (local representatives)	In-depth interviews, group discussion, self-administered questionnaires	Extreme weather—flood, storm
Wu et al. [19]	Qualitative description	2019	China	21	Professionals (researchers, physicians, local officers, dispatches rescue experts)	In-depth interviews, report and thematic analysis	Extreme weather—flood
Amstislavski et al. [24]	Correlational and time series analysis—quantitative study	2013	Russia	370	Locals (nomadic herders)	Clinical visits, record analysis, Spearman correlation—to measure the relation between temperature anomalies and the arrival of herders at Nes clinic. Piecewise regression	Meteorological—rise in temperature
Lindvall et al. [16]	Qualitative description	2020	Ethiopia Kenya Somalia	39	Professionals (United Nations (UN) agencies, government ministries, Non-Governmental Organizations (NGOs), and regional leaders)	In-depth interviews, workshop, review of the published literature	Extreme weather—drought
Haileselassie et al. [28]	Ecological study—quantitative study	2022	Ethiopia	N/A	No human participants	Report analysis on malaria morbidity, molarity and prevention, meteorological data collection, multivariate analysis	Meteorological—relative humidity, rainfall
Rosen et al. [32]	Qualitative description	2021	Zambia	181	Locals (adults and women in drought-affected areas), health experts, civic leaders	In-depth interviews, focus group discussions	Extreme weather—drought
Yiran et al. [33]	Qualitative description	2017	Ghana	13 communities were involved—the exact number of participants was not specified	Locals professionals (people with different backgrounds, ranging from those who have experienced at least one of the hazards and/or have knowledge of climate change, community leaders, rich/poor, professionals, and educated/uneducated)	In-depth interviews, Group discussions	General
Kendrovski et al. [17]	Vulnerability and adaptation assessment to climate change	2014	Macedonia	N/A	A group of climate and health experts, as well as representatives of NGOs and government agencies	Secondary research	General
Duarte et al. [29]	Ecological Quantitative study	2017	Brazil	N/A	No human participants	Data collection—data was retrieved from the national hospital Information System of Brazil (he SUS), National Institute of Meteorology and the National Water Agency Multiple Poisson and negative binomial regression models were used.	Extreme weather- Flood
Souza et al. [30]	Ecological quantitative study	2012	Brazil	N/A	No human participants	Data collection—daily data on admissions for respiratory diseases, precipitation, air temperature, humidity, and wind speed for the 2004–2008 period. Generalised linear models, with Poisson multiple regression	Meteorological changes in temperature, humidity, precipitation and wind speed
Ebi et al. [18]	Mixed-method study	2017	Albania, Barbados, Bhutan, China, Fiji, Jordan, Kazakhstan, Kenya, Kyrgyzstan, Philippines, Russian Federation, Tajikistan, and Uzbekistan	19	A group of climate and health experts, as well as representatives of NGOs and government agencies	In-depth interviews, focus groups, discussions, evaluating project reports of multinational health adaptation. project between 2008–2013	General
Leal Fet al. [31]	Comparative analysis—quantitative study	2018	Austria, Ethiopia, Malaysia, Uruguay, South-Eastern Austria, Douala, La Paz, Dar-es-Salaam	N/A	No human participants	Cross-comparison analysis and analysis	General

**Table A4:** MMAT—qualitative studies.

	Are there clear qualitative and quantitative research questions (or objectives *), or a clear mixed-methods question (or objective *)?	Do the collected data address the research question (objective)? E.g., consider whether the follow-up period is long enough for the outcome to occur (for longitudinal studies or study components)	Are the sources of qualitative data (archives, documents, informants, observations) relevant to the research question (objective)?	Is the process for analysing qualitative data relevant to the research question (objective)?	Has the researcher considered how the findings relate to the context, e.g., the setting, in which the data were Collected?	Do researchers take into account how their influence on participants may be affected by their findings?	Does coherence exist between qualitative data sources, collection, analysis, and interpretation?
Stewart-Ibarra et al. [13]	Yes	No	Yes	Yes	Yes	No	Yes
McMichael et al. [20]	Yes	Yes	Yes	Yes	Yes	yes	Yes
Abdullah et al. [50]	Yes	Yes	No	Yes	Yes	Yes	Yes
Kabir et al. [21]	Yes	Yes	Yes	Yes	Yes	No	Yes
Sahoo et al. [23]	Yes	Yes	Yes	Yes	Yes	No	Yes
Wu et al. [19]	Yes	Yes	no	Yes	Yes	No	Yes
Lindvall et al. [16]	Yes	No	Yes	Yes	Yes	No	Yes
Rosen et al. [32]	Yes	Yes	No	Yes	Yes	No	Yes
Yiran et al. [33]	Yes	Yes	Yes	Yes	Yes	No	Yes

**Table A5:** MMAT—Descriptive quantitative studies.

	Is the sampling strategy relevant to the research question?	Is the sample representative of the target population?	Are the measurements appropriate?	Is statistical analysis appropriate to answer the research question?
Lowe et al. [25]	Yes	Yes	Yes	Yes
Aik et al. [27]	Yes	Yes	Yes	Yes
Amstislavski et al. [24]	Yes	Yes	Yes	Yes
Haileselassie et al. [28]	Yes	Yes	Yes	yes
Malik S et al. [26]	Yes	Yes	Yes	Yes
Duarte et al. [29]	Yes	Yes	Yes	Yes
Souza et al. [30]	Yes	Yes	Yes	
Leal Fet al. [31]	Yes	Yes	Yes	Yes

**Table A6:** MMAT—mixed-method studies.

	Is there an adequate rationale for using a mixed-methods design to address the research question?	Are the different components of the study effectively integrated to answer the research question?	Are the outputs of the integration of qualitative and quantitative components adequately interpreted?	Are divergences and inconsistencies between quantitative and qualitative results adequately addressed?	Do the different components of the study adhere to the quality criteria of each of the methods involved?
Haque et al. [22]	Yes	Yes	Yes	No	Yes
Van Minh et al. [15]	Yes	Yes	Yes	No	Yes
Ebi et al. [18]	No	Yes	Yes	No	Yes
